# Assessing the learning curve for transumbilical single-site laparoscopy for endometrial cancer

**DOI:** 10.3389/fonc.2024.1337719

**Published:** 2024-02-01

**Authors:** Fanlin Li, Ying Zheng, Fan Yang, Jianhong Liu

**Affiliations:** Department of Gynecologic Oncology, West China Second Hospital, Sichuan University, and Key Laboratory of Birth Defects and Related Diseases of Women and Children (Sichuan University), Ministry of Education, Chengdu, Sichuan, China

**Keywords:** transumbilical laparoendoscopic single-site surgery, minimally invasive surgery, endometrial cancer, learning curve, CUSUM

## Abstract

**Introduction:**

Applying transumbilical laparoendoscopic single-site surgery to endometrial cancers is worldwide, and the depiction of the learning curve is rarely described, which leads to the vagueness of young clinical practitioners. We accumulated the data to identify the completion of the learning curve by analyzing the operative and postoperative outcomes of the patients with endometrial cancer for transumbilical laparoendoscopic single-site surgery (TU-LESS).

**Methods:**

This was a retrospective, consecutive single-center study of patients with endometrial cancer undergoing standard endometrial cancer comprehensive staging surgery (extrafascial hysterectomy, bilateral salpingectomy, and pelvic lymphadenectomy) through TU-LESS by an experienced surgeon from December, 2017 to June, 2021 in the Department of Gynecologic Oncology, West China Second Hospital, Sichuan University, China.

**Results:**

After applying the inclusion and exclusion criteria, 42 patients were included in the study. The learning curve for this study was evaluated using both cumulative sum (CUSUM) and risk-adjusted CUSUM (RA-CUSUM) methods. Applying CUSUM and RA-CUSUM has grouped 42 cases into three phases. The prior five cases represented the learning period. The following six cases were needed to lay a technical foundation (cases 6–11). The third phase was regarded as achieving proficiency (cases 12–42). The operative time decreased drastically with the learning curve. There were no significant differences in terms of postoperative complications and lymph node retrieval among the three phases. More difficult patients were confronted in the third phase.

**Discussion:**

In our study, the learning curve was composed of three phases. According to the results of our study, 11 cases were required for experienced surgeons to achieve a technical foundation.

## Introduction

Endometrial cancer is the sixth most common cancer among women globally (1, 2). High-income countries exhibit a higher incidence of this malignant disorder, with 11.1 cases per 100,000 women (2). The incidence is still increasing (3). As endometrial cancer is frequently diagnosed at an early stage, an ideal outcome is expected with early medical intervention (4). Conventional laparoscopy is a widely recommended and accepted treatment modality as it achieves the same therapeutic results and causes less trauma to the patients compared to laparotomy (4). Transumbilical laparoendoscopic single-site surgery (TU-LESS) is an emerging technique that is the least minimally invasive substitution for conventional laparoscopy (5, 6). It is superior as it results in faster patient recovery and causes less pain, which shortens the time window before patients receive adjuvant therapy (7). Various clinical trials have verified its safety in terms of oncological outcomes (8). Previous reports have also demonstrated that a better subjective cosmetic result was obtained with TU-LESS (9, 10).

TU-LESS is performed through one incision. Hence, the surgeons may have trouble manipulating instruments (11, 12). Due to this, the procedure is likely to induce fatigue and is challenging to master. The learning curve can vary massively for different surgeons. Herein, we have presented the learning curve analysis of the 3-year cumulative experience of one surgeon at our institution for applying TU-LESS in endometrial cancer. This was done by analyzing the perioperative outcomes of patients with endometrial carcinoma undergoing TU-LESS.

## Materials and methods

### Patient population

From December, 2017 to June, 2021, a series of consecutive 71 endometrial cancer cases who experienced TU-LESS were to be performed by a single surgeon accompanied by experienced assistants at the Department of Gynecologic Oncology, West China Second Hospital, Sichuan University, China. To evaluate the surgical outcomes, 42 cases who went through extrafascial hysterectomy, bilateral salpingectomy (BSO), and pelvic lymphadenectomy were elected. Due to different surgeries, 29 cases were excluded. We examined the age, underlying disease, gestation and pregnancies, and family history of patients as background data. All patients were informed about the procedures, the advantages of TU-LESS, and the potential risks. Institutional Review Board approval was obtained.

### Data collection and definitions

All data were retrieved from West China Second Hospital, which were investigated and retrospectively viewed.

The data evaluated included patients’ demographics, operative variables, and postoperative data.

Patient demographics include age, BMI, childbearing history, the presence of medical and surgical comorbidities, carbohydrate antigen 125 (CA 125), and carbohydrate antigen 19-9 (CA 19-9).

Operative variables studied included operative time, estimated blood loss, conversions to other techniques, and intraoperative complications.

Postoperative data included VAS score, hospital stay, time to first passage of flatus, postoperative complications, and pathology results.

The primary endpoint was operative time, which was used for cumulative sum (CUSUM) analysis. Operative time was identified as the duration from the first incision to the final closure. The second endpoints were conversions and short-term complications, which were used for the risk-adjusted CUSUM (RA-CUSUM) analysis. The pain score was acquired 12 h, 24 h, and 36 h after surgery, and we marked the according intervention and outcomes. Patients met the discharge standard if their temperature was normal, the catheter was removed with unblocked urination, and the first passage of the flatus was done without any abnormal complications or laboratory tests, which time frame was set as the enhanced recovery index (ERI). The hospital stay was identified as the interval from the surgery to the day of discharge.

Short-term complications were stratified in accordance with the Clavien–Dindo classification of surgical complications, which was applied to assess the success of surgeries. The pathological results and staging status were recorded based on the International Federation of Gynecology Oncology. We demanded patients be reviewed in outpatient clinics regularly.

### Surgical procedure

The patient undergoes a thorough cleaning and sterilization procedure at the umbilicus 24 h before the operation. Preoperative administration of antibiotics is conducted 30 min before the surgical procedure to decrease the risk of infection. The procedure is conducted using general anesthesia. Following the administration of anesthesia, the patients had standard disinfection using a towel, and a urinary catheter was inserted. Additionally, the assistants perform a resterilization procedure on the vulva and vagina.

Following the sterilization, the assistant inserts a simple uterine manipulator, which facilitates visibility of the surgical area. The patients were positioned supine, with the head lowered and feet elevated, throughout the procedure. Available visualization equipment options include the Olympus or the German STORZ laparoscopic system.

The traditional laparoscopic instruments include separation forceps, non-invasive grasping forceps, suction, scissors, curved forceps, needle holders, and others. The extended instruments consist of all 45-cm extended needle holders, suction, non-injury grasping forceps, and others. Additionally, energy devices are also used. The energy instruments employed included the Johnson & Johnson Harmonic ultrasonic knife, an extended 45-cm ultrasonic knife, single and double electrocoagulation forceps, and BiClamp. The Johnson & Johnson SXPP1B401 barbed wire was utilized for vaginal suturing in the study.

Port preparation involves using a Kangji disposable single port, which has one inlet with a diameter of 10 mm, another with a diameter of 12 mm, and two inlets with a diameter of 5 mm each.

**Figure 1:**
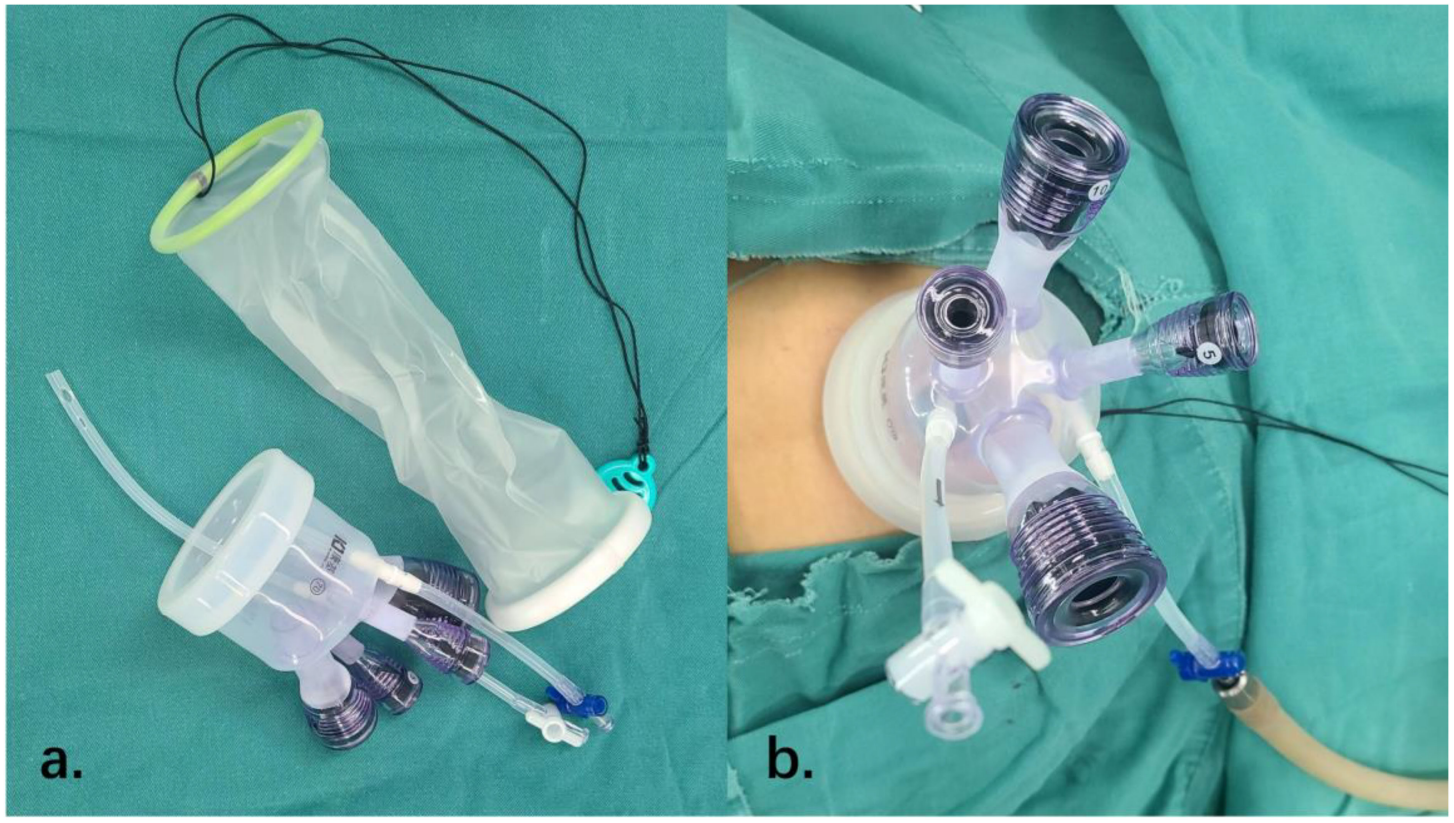
The establishment of transumbilical single-site laparoscopy. **(A)** The incision protective sleeve and the port we used to perform the surgery. **(B)** The establishment of transumbilical single-site laparoscopy.

As for medical operations, we perform umbilicus sterilization, create a 2-cm incision in the center of the umbilicus aligned with the body’s longitudinal axis, and sequentially cut through the layers of skin, where a port is inserted (**Figure 1**).

Following the creation of a pneumoperitoneum, a laparoscopic lens and surgical equipment are introduced into the port. A comprehensive examination of the pelvic and abdominal regions is conducted, and fluid from the pelvic and abdominal lavages is collected for analysis.

Additionally, the fallopian tubes are treated before the surgery. The surgery involves the removal of one or both fallopian tubes or ovaries, the complete removal of the uterus (the extent of which will be determined based on preoperative staging and intraoperative circumstances), and the surgical removal of lymph nodes. The “Zheng’s 3C suspension method” is employed to aid with intraoperative exposure for individuals facing challenges in this regard (13).

Following the procedure, a T-tube is inserted into the pelvic floor to facilitate drainage of the vaginal canal, while the vaginal incision is sutured using barbed wire.

The umbilical incision is closed by applying traction to the apical line of the peritoneofascial incision, securing it with knots, anchoring it, and then shaping and beveling it to ensure proper closure. This process requires specialized techniques, which we invented and named “anchoring technique” (14). At last, we apply gauze to compress the umbilicus and use disposable dressings to recover a natural appearance.

### Evaluation of surgical performance

All procedures were performed by one skilled surgeon who had accumulated many experiences in conventional laparoscopy applied to malignant diseases and TU-LESS treating gynecological benign lesions. In order to identify surgical results, patient demographics, operative variables, and postoperative data were investigated.

### Statistical analysis

All statistical analyses were performed using SPSS version R26.0.0.0 and SAS^@^ studio. Normally distributed continuous variables were presented as mean and standard deviation. Categoric variables were exhibited as frequency. Differences in characteristics among groups were analyzed using the Chi-test with *post hoc* tests or Fisher’s exact test (for categorical variables) and Bonferroni *t*-test (for continuous variables). Logistic regression was used to assess the second turning point of the learning curve. A *p*-value of <0.05 was considered statistically significant.

### CUSUM analysis

We used CUSUM analysis to determine which point was defined as the completion of the learning curve. CUSUM is widely accepted to evaluate the learning curve and distinguish the learning, proficient, and mastering phases (15). In this study, we calculated the CUSUM by ranking the cases chronologically from the first to the latest date of endometrial cancer using TU-LESS. CUSUM (1) = the first operation time OT (1) − the average operation time OT (mean), CUSUM (*n*) = OT (*n*) − OT (mean) + CUSUM (*n*−1), until the last CUSUM was calculated as 0. Furthermore, we established a trend line to show the change in the slope of the learning curve, based on which the inflection points were identified. The ascending phase of the trend line indicates it is located in the primary learning phase of this technique. The first inflection point demonstrated the completion of this procedure. The descending line showed the surgeon had laid a technical foundation.

### RA-CUSUM analysis

RA-CUSUM analysis was used to depict the success or failure of this technique. It was recognized as an extension of CUSUM to further assess the learning curve. In order to define the failure of surgery, we selected three parameters, including conversions, postoperative complications (Clavien ≥ III), and 30-day readmission. Any occurrence of one of these three events was defined as a surgical failure.

Univariate analysis was applied to analyze the risk factors, including all perioperative data and pathological results except the three events above. RA-CUSUM was defined as RA-CUSUM*∑^n^_i_*_= 1_(*x^i^*−*τ*) + (−1)*^x^* ^i^P*_i_*. We used *x_i_* = 1 to symbolize the presence of surgical failure, and *τ* represents the observed event rate. The expected rate is *P_i_*, which is retrieved through the regression model. Therefore, the descending line refers to surgical success and the ascending line to surgical failure. However, due to the small sample size, we failed to filter multiple positive indexes to proceed with the calculation, so we incorporated operation time, surgical comorbidities, lymph nodes, and exhaust time, which were commonly seen as factors to influence the outcome and statistical analysis, all to finish the simulation.

## Results

### Patients’ characteristics and surgical outcomes

We viewed 42 endometrial cancers between December, 2017 and June, 2021, with two conversions to porous laparoscopy in the second phase (**Table 1**). As to the pathologic results, after total clinical staging, there were 35 IA, four IB, two IIIA, and one IIIC. The average age for patients was 46.74 (SD = 10.33). The mean BMI is 24.62 (SD = 4.02) kg/m^2^; 14 (33%) patients had a history of previous pelvic and abdominal surgery. The average operating time and blood loss were 207.45 (SD = 40.64) min and 97.38 (SD = 85.69) ml, respectively. The average time for the first passage of flatus was 2.57 (SD = 0.89) days. The hospital stay was 5.05 (SD = 1.36) days. The enhanced recovery index (ERI) was 2.97 (SD = 0.92) days. The reasons for two conversions to multiport laparoscopy were severe adhesion in the pelvic cavity and injury to the external iliac vein because of obesity, with extreme difficulty in exposing the field. Two cases with failed sentinel lymph node mapping were reported, which required a change of operation from resection of sentinel lymph nodes to lymphadenectomy. One lymphatic retention with infection appeared. After four phases of follow-up, only two patients reported successively observed lymphatic cysts by radiology, and they have been constantly monitored with no evidence suggesting a relapse ever since. No more late complications are observed.

**Table 1:** Preoperative parameters of three stages for TU-LESS in endometrial cancer (n = 42).

	Total	First group (*n* = 5)	Second group (*n* = 6)	Third group (*n* = 31)	*p*-value	*p*1	*p*2	*p*3
Demographics								
Age	48.12 (10.30)	46.80 (10.05)	45.33 (7.09)	48.87 (10.99)	0.720	0.818	0.453	0.684
Manifestations								
Irregular vaginal bleeding	31	3	5	23				
Excessive menstruation	7	0	1	6				
Vagina discharge	3	1	0	2				
Infertility	1	1	0	0				
Medical comorbidity								
History of cancer	2	0	1	1				
Hypertension	6	0	0	6				
Diabetes	2	0	1	1				
PCOS	1	1	0	0				
Infertility	1	1	0	0				
Surgical comorbidity	0.57 (0.74)	0.20 (0.45)	0.83 (1.17)	0.58 (0.67)	0.371	0.164	0.447	0.290
Laparotomy	12	0	3	11				
Laparoscopy	2	1	1	1				
Menopause								
Yes	11	2	1	8	0.423	0.317	0.405	0.132
No	31	3	5	23				
Childbearing history								
Gestation	2.83 (2.26)	3.20 (2.59)	3.33 (2.50)	2.66 (2.22)	0.749	0.924	0.516	0.628
Pregnancy	1.18 (1.03)	1.2 (1.10)	1.33 (0.82)	1.13 (1.09)	0.918	0.837	0.683	0.904
Family history	15	2	2	11	0.529	0.655	0.071	0.033
BMI	24.62 (3.89)	23.17 (3.41)	25.32 (3.57)	24.73 (4.07)	0.645	0.373	0.737	0.418
Preoperative histology								
Endometrioid G1	7	1	0	6				
Endometrioid G2	6	0	2	4				
Endometrioid G3	5	0	0	5				
Dysplasia	12	4	2	6				
Serous	1	0	0	1				
Mucinous	2	1	0	1				
Carcinosarcoma	1	0	0	1				
Mixed	2	1	0	1				
Unidentified	8	0	2	6				

### Assessment of creating a learning curve

**Figure 2:**
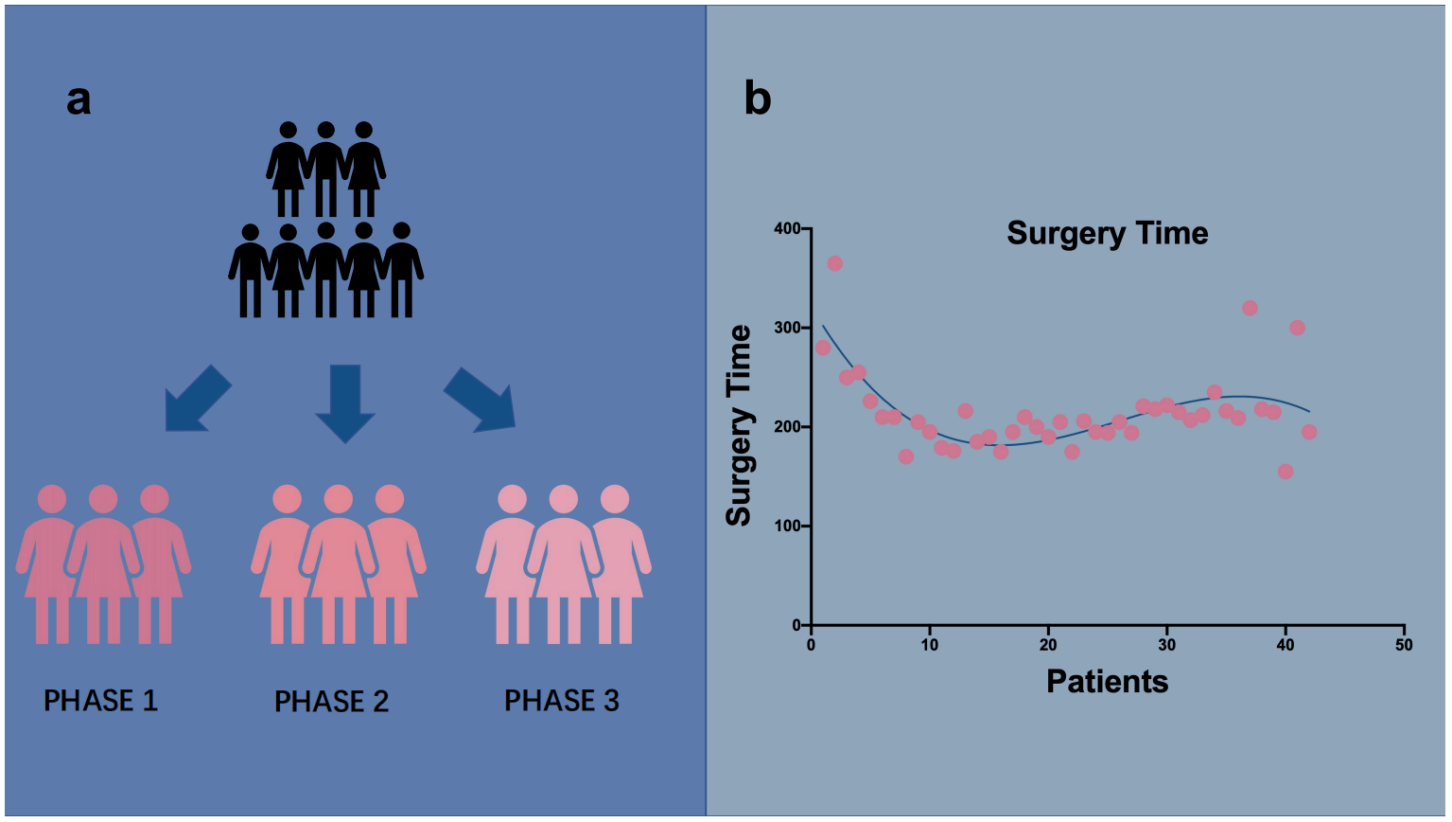
Group status and linear graph of operation time. **(A)** We separated patients in three groups In chronological order. **(B)** Surgery time is displayed in b which showed a declining trend.

Our learning curve is exhibited in **Figure 2**.

**Figure 3:**
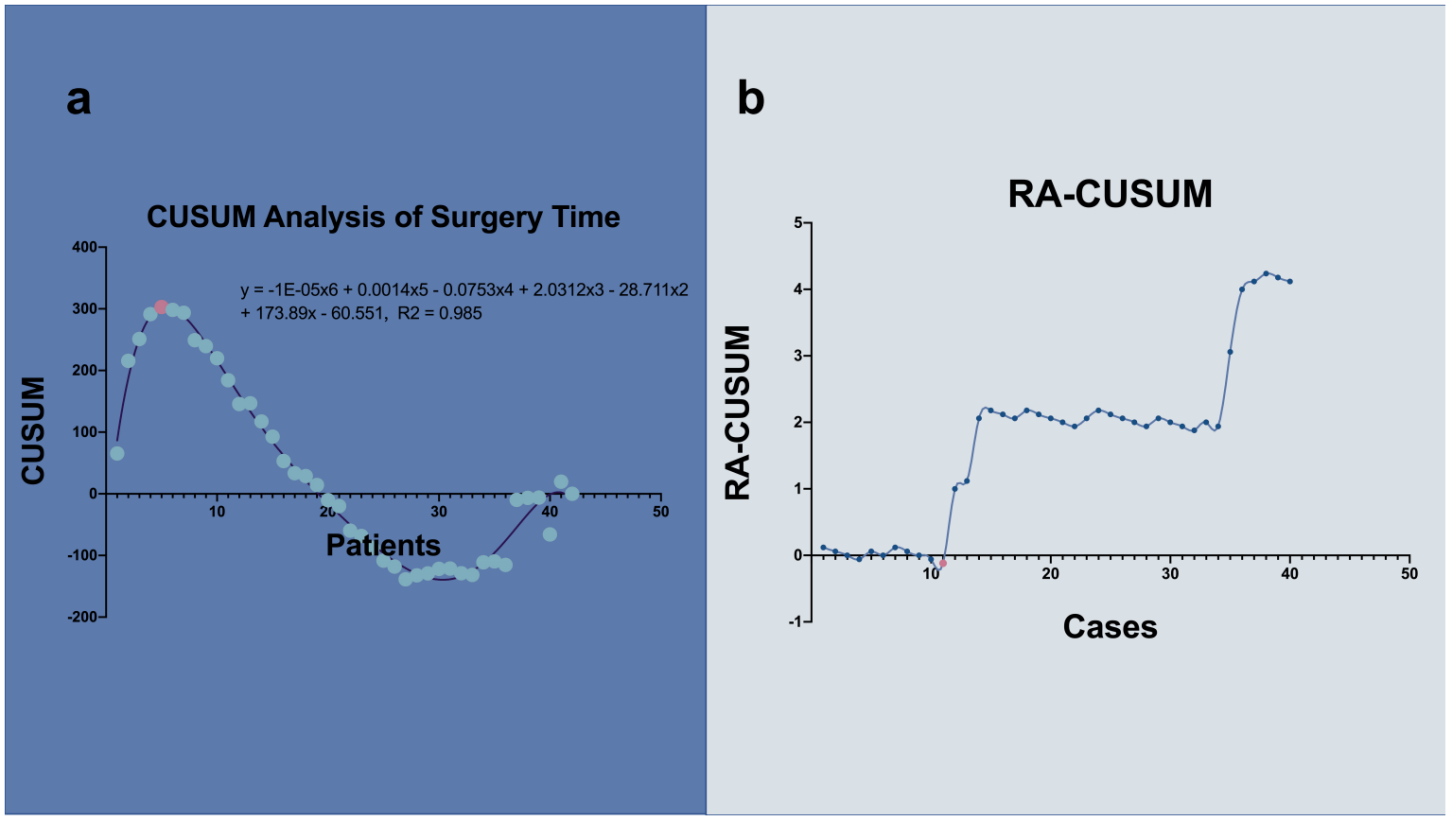
CUSUM and RA-CUSUM analysis of learning curve. **(A)** The inflection point separated the patients into 2 phases. The purple line represents the curve of best fit in a general model with equation, y = -1E-05x6 + 0.0018x5 - 0.091x4 + 2.3675x3 - 32.536x2 + 204.23x - 115.01, R2 = 0.9658. **(B)** The valley point divided the curve into 2 phases. 5 cases are needed to lay solid foundation and 11 to acquire proficiency. Corresponding points were marked with light pink.

The first inflection was in five surgical cases (**Figure 2**). Two phases were primarily differentiated on the graph. Although the operation time tended to decrease after five cases, the descent time did not imply competence in TU-LESS. RA-CUSUM was introduced to further assess the learning curve (**Figure 3**). The three parameters were imported. According to the RA-CUSUM graph, the valley point was presented in the 11th case with minimal surgical failure, which was considered the achievement of competence. Combining the results of these two methods, the learning curve for TU-LESS in treating endometrial cancer was divided into three groups. The first group (cases 1–5) represented the initial learning period. The second group, which spanned six cases (cases 6–11), indicated the developed competence. The last group signified mastery and a challenging period.

### Assessment of the credibility according to the learning curve

As the inflection point was between five and 11 cases, patient backgrounds, preoperative pathophysiology, surgical results, and intraoperative and postoperative outcomes were examined and compared by dividing the patients into three phases. Each phase was analyzed for the potential learning curve effect. The baseline conditions were compared, and the three phases did not show significant differences. The mean operative time varied among the three phases. In the first two phases, it showed a drastic reduction. However, the third phase demonstrated an upward trend, exhibiting 275.20 (SD = 53.74) min, 194.83 (SD = 16.92) min, and 208.68 (SD = 31.99) min, respectively (**Table 2**). The average surgical estimated blood loss was 94 ml less, comparing 153.00 (SD = 153.56) ml to 60.00 (SD = 43.82) ml in the first two phases. No major intraoperative complications occurred, and only two cases were converted from single port to conventional laparoscopy due to severe pelvic adhesion. Postoperative outcomes did not show significant improvement over time among the three phases. No hospital readmission was observed in both groups for medical or surgical complications. Pathologic details were displayed in (**Table 3**).

**Table 2:** Perioperative surgical outcomes of three stages for TU-LESS in endometrial cancer (n = 42).

Operative outcomes								
	total	1st group (n=5)	2nd group (n=6)	3rd group (n=31)	*P*-value	P1	P2	P3
Surgical time	214.62 (39.96)	275.20 (53.74)	194.83 (16.92)	208.68 (31.99)	.000	.000	.000	.359
Blood loss	132.02 (203.85)	154.00 (153.56)	60.00 (43.82)	142.42 (228.25)	.653	.457	.377	.908
Surgical complications								
Vascular complication	3	0	0	3	.392		.317	.480
Perioperative outcomes								
Postoperative complications					.752			
Lymphatic retention with infection	1	0	0	1				
Sentinel lymph node	2	0	0	2				
Conversion	2	0	0	2				
Hospital stay	5.05 (1.36)	4.40 (5.55)	4.83 (1.17)	5.19 (4.47)	.452	.872	.840	.490
Enhanced recovery index	2.97 (0.92)	2.60 (0.89)	2.67 (0.52)	3.13 (0.99)	.331	.151	.041	.533
First passage of flatus	2.57 (0.89)	3.00 (0.71)	2.50 (0.84)	2.52 (0.97)	.527	.656	.999	.538
VAS pain score								
12		2.2 (0.84)	1.67 (0.82)	1.79 (0.82)	.470			
24		1.60 (0.55)	2.17 (0.41)	1.66 (0.55)	.115			
36		0.80 (0.45)	0.50 (0.84)	1.17 (0.71)	.084			

**Table 3:** Oncology outcomes of three stages for TU-LESS in endometrial cancer (n = 42)..

Oncology outcomes								
	total	1st group (n=5)	2nd group (n=6)	3rd group (n=31)	P value	P1	P2	P3
I								
IA	27	1	5	18				
IB	2	0	0	2				
IA, G2-3, serous, mucinous, clear	9	3	2	7				
IB, G2-3, serous, mucinous, clear	1	0	0	1				
III								
IIIA	2	1	1	0				
IIIC	1	0	0	1				
Pathology details								
Peritoneal wash								
Positive	2	0	0	2	.540		.157	.257
Negative	34	3	6	25				
NE	6	2	0	4				
LVSI	10	0	0	10	.030		.127	.174
Number of pelvic lymph nodes	28.19 (10.10)	25.6 (13.35)	23.67 (5.13)	29.48 (10.21)	.369	.753	.204	.429
Adjuvant therapy								
Chemotherapy	4	0	1	3				
Radiotherapy	3	0	0	3				
Chemotherapy and radiotherapy	2	0	0	2				

## Discussion

TU-LESS is now being increasingly utilized to treat early endometrial cancer (8). A surgeon’s learning curve provides us with a retrospective view of their performance. Although previous studies have assessed the learning curves of TU-LESS in endometrial cancer, they were restricted by simply arranging chronological cases into predefined segments (16). In the present study, we investigated 42 cases of endometrial cancer with LESS performed by a single surgeon at Sichuan University Second Hospital. We used the CUSUM and RA-CUSUM methods (**Figures 2**, **3**) to evaluate our learning curve. The efficacy of this technique was achieved after five surgical cases.

TU-LESS was first widely utilized in urology and gastrointestinal surgery (17–19), where it was reported that surgeons entered their proficient stage after performing 30 cases (20). A previous study on endometrial cancer has described a learning curve with proficiency achievement after 20–40 cases (16).

It should be emphasized that our surgeon had performed over 53 procedures using TU-LESS for benign lesions and had already mastered surgical treatments for malignant tumors using conventional laparoscopy before conducting comprehensive staging surgery for endometrial cancer through TU-LESS. This could be why our surgeon only required five surgical cases to lay the technical foundation.

However, after the valley point in the RA-CUSUM graph, there was a trend of increasing surgical failure, which could be attributed to the surgeon operating on more challenging patients with higher risks.

Our surgeon required 11 cases to master this technique, a finding that is different from those of previous studies (21, 22). There was a significant reduction in operative time in the three groups. Pelvic lymph node retrieval and perioperative complications did not demonstrate significant differences. This result was observed because the procedure had been standardized and the baseline of the patients remained consistent.

Barnes et al. reported after including 110 patients that the average surgery time was 186 min (16) compared to the 208 min observed in our study. This could be due to the different operation modes in every institution and the unique anchor-suturing method (14) technique we applied. The difference was deemed acceptable.

During further exploration, we noticed that the experience of the assisting team, especially the assistant holding the laparoscope, could distort the vision, leading to disorientation. This could cause difficulty in identifying lesions, leading to prolonged operation time, potential injury to the vasculature, inadequate resection, etc. Our team had mastered laparoscopy with a relatively fixed and coordinated assistant.

Applying LESS for endometrial cancer is relatively easy for skilled operators. However, different operators may have entirely different learning curves because of their unique inline vision and relatively narrow space to operate instruments, including the difficulty of the operating handles in forming triangulations. With the change of approach, the position and postures of practitioners and the different angles of holding the instruments also vary from conventional laparoscopy. Various levels of adaptation add up to the diversity of the learning curves for different surgeons.

LESS for endometrial cancer is an advanced technique that requires the operator to be familiar with the anatomy of the pelvic cavity and proficient with laparoscopic manipulation. The learning curve is an individualistic study. We have concluded that beginners should start with simple surgeries, such as appendectomies and hysterectomies, to accumulate experience and skills. Beginners should skip ovarian cyst excisions at the start since any procedure involving suturing beneath the magnifier would enhance the difficulty.

This study might have limitations, such as a small number of patients, the insignificance of statistical results, and the absence of long-term results. It is essential to explore whether oncological outcomes might be compromised during the primary learning period. It was inevitable that the study population would be restricted to a smaller size. However, this study was an individualistic study to provide our experience in shortening the learning curve. Since combining the experiences of other practitioners in the same field could improve surgical outcomes, more studies from multiple centers should be carried out to help accumulate experience. Meanwhile, due to the early diagnosis and relatively good prognosis, quality of life and sexual function are emerging demands for patients receiving surgical intervention, which were previously underappreciated (23). We could expand on our findings by looking into this novel topic further.

Despite many obstacles, the emergence of robotic-assisted single-site laparoscopy has been advantageous (24, 25). It has been reported that the conversion rate could significantly decrease, and learning curves can be shortened with the assistance of a robot (26, 27). As stated earlier, we believe it is easy for experienced surgeons to master this technique. Our surgeon, who had excellent laparoscopy experience, mastered this technique after completing five endometrial cancer comprehensive staging operations with TU-LESS.

## Data availability statement

The original contributions presented in the study are included in the article/supplementary material. Further inquiries can be directed to the corresponding author.

## Ethics statement

The studies involving humans were approved by West China Second Hospital Ethics Committee. The studies were conducted in accordance with the local legislation and institutional requirements. Written informed consent for participation was not required from the participants or the participants’ legal guardians/next of kin because this was a retrospective study. This study was conducted after the patient was discharged. Written informed consent was not obtained from the individual(s) for the publication of any potentially identifiable images or data included in this article because This was a retrospective study, and when this study was conducted, the patient was discharged.

## Author contributions

FL: Data curation, Formal analysis, Investigation, Methodology, Writing – original draft. YZ: Funding acquisition, Resources, Visualization, Writing – review & editing. FY: Investigation, Supervision, Writing – review & editing. JL: Project administration, Writing – review & editing.
